# Delayed fixation of neglected acetabular fractures: clinical challenges and mid-term outcomes of a prospective case series

**DOI:** 10.1186/s13018-025-06298-7

**Published:** 2025-10-08

**Authors:** Mahmoud Fahmy, Hazem Abdelazeem, Ahmed Hazem Abdelazeem, Mostafa Ahmed Shawky

**Affiliations:** Pelvis Fracture and Arthroplasty Unit, Orthopaedic Department, Kasr Alainy Hospital, Cairo University, Cairo, Egypt

**Keywords:** Neglected acetabular fractures, Open reduction and internal fixation, Delayed surgery

## Abstract

**Objective:**

Neglected acetabular fractures, defined as those not addressed surgically within three weeks of injury, pose significant technical and prognostic challenges due to chronic displacement, malunion, and soft tissue contracture. This study aimed to evaluate radiological and functional outcomes of open reduction and internal fixation (ORIF) in a large prospective cohort managed at a tertiary trauma center.

**Methods:**

Between 2009 and 2019, patients aged 14 years and older with displaced, neglected acetabular fractures (> 3 weeks post-injury) were enrolled at a high-volume Level I pelvic trauma center. Exclusion criteria included pathological fractures, fresh fractures, unreconstructable articular surfaces, and patients who did not complete the minimum follow-up period. Of the total cohort, 94 patients underwent ORIF and were analyzed. Preoperative evaluation included CT imaging, and fractures were classified using the Letournel–Judet system. Radiological reduction quality was graded by Matta’s criteria, and functional recovery was assessed using the modified Merle d’Aubigné–Postel score. Secondary outcomes included complication rates and conversion to THA. For subgroup analysis, patients were stratified into early-late presenters (3–5 weeks) and very-late presenters (> 5 weeks). Statistical analysis employed chi-square tests for categorical variables, Student’s t-test or Mann–Whitney U test for continuous variables, and multivariate logistic regression to identify predictors of outcome, with significance set at *p* < 0.05.

**Results:**

Among the 94 patients analyzed, anatomical reduction (≤ 1 mm displacement) was achieved in 59.6%, with imperfect and poor reductions in 26.6% and 13.8%, respectively. Quality of reduction (β = 0.44, *p* < 0.001) and surgical delay (β = − 0.32, *p* = 0.006) independently predicted functional outcome, while fracture type and femoral head condition were not significant.On subgroup comparison, early-late presenters (3–5 weeks) showed a trend toward better outcomes compared with very-late presenters (> 5 weeks), although differences did not reach statistical significance. Major complications included heterotopic ossification (12.8%), post-traumatic arthritis (14.9%), avascular necrosis (9.6%), and conversion to THA during follow-up (10.6%).

**Conclusion:**

This study represents one of the largest prospective series on neglected acetabular fractures with midterm follow-up. Despite delayed presentation, ORIF achieved satisfactory anatomical and functional results in most cases. Early surgical intervention within the neglected window was associated with superior outcomes, while subgroup analysis highlighted a gradual adverse effect of increasing delay. These findings underscore the importance of individualized surgical strategies, meticulous planning, and experienced surgical execution in optimizing results for this challenging patient population.

## Introduction

Acetabular fractures rank among the most technically demanding injuries in orthopedic trauma due to the complex pelvic anatomy and the pivotal role of the hip joint in mobility and weight-bearing. Optimal outcomes are closely linked to early surgical management, ideally within three weeks of injury, which maximizes the likelihood of achieving anatomical reduction and reduces complications such as avascular necrosis (AVN), heterotopic ossification (HO), and post-traumatic arthritis [1].

In many resource-limited settings, however, a substantial proportion of patients present late. Neglected acetabular fractures, commonly defined as those treated more than 21 days after injury, represent a particularly challenging subgroup. Contributing factors include delayed diagnosis, lack of specialized trauma services, limited access to advanced imaging, and patient-related barriers such as comorbidities or financial constraints [1, 2]. With increasing chronicity, fibrosis, callus formation, and malunion make surgical reduction progressively more difficult, while posterior wall fractures may undergo resorption or AVN, further restricting reconstructive options [2, 3].

Although retrospective series suggest that open reduction and internal fixation (ORIF) may still provide acceptable outcomes in delayed cases, the available evidence remains limited, heterogeneous, and often lacks prospective midterm follow-up [2]. The prognostic impact of surgical delay, fracture pattern, and patient-related variables on both radiological and functional outcomes is not fully defined.

To date, no large prospective cohorts have systematically evaluated the outcomes of ORIF in neglected acetabular fractures. In particular, the effect of surgical delay within the neglected window, alongside other prognostic factors, on radiological reduction, functional recovery, and complication rates has not been adequately quantified.

This prospective cohort study aimed to evaluate radiological and functional outcomes of ORIF in neglected acetabular fractures with a surgical delay beyond 21 days. We hypothesized that ORIF remains a feasible and effective reconstructive option in selected delayed presentations, and that shorter surgical delay within the neglected window is independently associated with superior anatomical reduction and functional outcomes. In addition, patient- and fracture-related variables (fracture pattern, femoral head viability, surgical approach) were expected to further influence prognosis. By clarifying these endpoints, the study seeks to provide evidence to guide surgical decision-making and optimize outcomes in late-presenting acetabular fractures.

## Patients and methods

Between January 2009 and January 2019, a prospective cohort study was conducted at University Hospital, a Level I pelvic trauma center, following approval by the Institutional Ethics Committee before the study starts. All patients meeting the eligibility criteria during the study period were enrolled consecutively to minimize selection bias. Recruitment was prospective, with standardized documentation of baseline demographics, fracture characteristics, and treatment details.

Consecutive patients aged 14 years and older who presented with displaced, neglected acetabular fractures more than three weeks after injury were eligible. To address skeletal maturity, pelvic radiographs were reviewed in all patients at admission. Individuals with open physes or incomplete acetabular ossification were excluded. The single 14-year-old patient included in this series demonstrated radiographic skeletal maturity and was managed with adult fixation principles.

Exclusion criteria were fresh fractures presenting within three weeks of injury, patients who did not complete the minimum follow up of 2 years, pathological fractures, and unreconstructable articular surface comminution, defined as multifragmentary collapse of the acetabular dome with loss of articular surface continuity or absence of cortical references that precluded anatomical reconstruction.

To clarify, this criterion referred specifically to cases in which stable fixation was technically unfeasible. Fractures with comminution that remained reconstructable—for example, both-column fractures with multiple fragments but with an intact or restorable acetabular roof—were included and managed with ORIF. Cases meeting exclusion criteria were treated with arthroplasty or salvage procedures outside the study cohort. All patients provided written informed consent at enrollment.

Causes of delayed presentation included missed diagnosis in polytrauma settings, inappropriate conservative treatment, misinterpretation of radiographs after closed reduction, failure of prior surgical intervention, or poor compliance with follow-up. Most patients presented with pain, inability to bear weight, deformity, limb shortening, or joint stiffness, or were referred following stabilization of more urgent injuries.

A standardized registry-based form was used for prospective data collection, capturing demographic information, comorbidities, mechanism of injury, associated injuries, baseline function, fracture pattern, treatment details, and outcomes.

All patients underwent focused neurological assessment and structured imaging protocol, including anteroposterior pelvic radiographs, Judet oblique views, and thin-slice CT (2 mm) for three-dimensional evaluation of fracture configuration, chronicity, and joint congruity. Fractures were classified according to the Letournel–Judet system. In patients with associated long-bone fractures, fixation of the limb injury preceded acetabular surgery whenever possible.

Surgery was performed by a dedicated pelvic trauma unit led by two senior orthopedic trauma surgeons with more than 10 years of experience in acetabular reconstruction. Residents and fellows assisted in exposure and fixation, but the key steps of reduction and implant placement were performed or directly supervised by the senior surgeons, ensuring consistency of technique.

The surgical goal was anatomical reconstruction of the acetabular dome and columns and restoration of hip joint stability. In all suitable cases, open reduction and internal fixation (ORIF) was performed. Arthroplasty or salvage procedures were reserved for unreconstructable fractures or those with avascular changes.

The surgical approach was individualized to fracture pattern and chronicity. For complex or both-column fractures, dual approaches were employed, often using a “floppy lateral” position with sequential repositioning into prone and supine postures for staged exposure. This facilitated stepwise adhesion release, fibrous callus removal, and mobilization of displaced fragments.

In severely neglected cases with dense fibrous contracture and non-mobilizable posterior column fragments, adjunctive maneuvers were occasionally required. These included ischial spine osteotomy to release the sacrospinous ligament or partial release of the sacrotuberous ligament. Such steps were undertaken only after conventional mobilization with traction, sequential soft-tissue release, and distraction–compression devices had failed. The intraoperative threshold was defined as persistent posterior column rigidity preventing anatomical reduction despite standard techniques. Risks of neurovascular injury, pelvic instability, and bleeding were carefully explained during preoperative counseling, and specific consent was obtained. No case developed instability or long-term morbidity attributable to these adjunctive procedures.

Fracture fragments were mobilized and reduced under direct vision with traction, clamps, and provisional K-wires. Reduction was verified fluoroscopically. Definitive fixation was achieved using low-profile reconstruction plates, contoured pelvic plates, and, where necessary, buttress plates or spring hooks. Operative time, blood loss, and intraoperative complications were recorded.

The primary exposure of interest was surgical delay, defined as the interval in days from the date of injury to definitive surgery. Because all patients presented after 3 weeks, instead, for secondary analysis, the cohort was stratified into two predefined subgroups: Early-late presentation: 3–5 weeks after injury, Very-late presentation: > 5 weeks after injury.This allowed comparison between moderately versus markedly delayed cases, while remaining consistent with the study’s original inclusion criteria.

Radiological outcomes were assessed postoperatively using AP and Judet views supplemented by CT to confirm reduction quality. Reductions were graded according to Matta’s criteria: anatomical (≤ 1 mm), imperfect (2–3 mm), or poor (> 3 mm).

Functional outcomes were assessed using the modified Merle d’Aubigné and Postel score at each follow-up visit. Patients who underwent conversion to THA were analyzed separately. In the main analysis, they were categorized as “poor outcome” to reflect treatment failure of ORIF. To test the robustness of this assumption, a sensitivity analysis was also performed in which pre-THA functional scores were retained in the dataset. The distribution of outcomes and the associations with reduction quality and surgical delay were compared between both approaches. Pre-THA functional scores were retained in the analysis; this did not materially alter the overall distribution or the statistical associations.

All radiological and functional outcomes were independently assessed by two pelvic trauma surgeons not involved in the index surgeries and blinded to patient identifiers and clinical data. Interobserver reliability was substantial for Matta’s grading (κ = 0.72) and excellent for the Merle d’Aubigné and Postel score (ICC = 0.84). Disagreements were resolved by consensus.

Radiographic healing was defined as continuity of cortical margins across the fracture line, absence of progressive displacement on sequential radiographs, and trabecular bridging on CT. Given the intra-articular nature of acetabular fractures, emphasis was placed on restoration and maintenance of joint congruity rather than conventional long-bone definitions of union. Radiographic union was not assessed for patients treated with conversion THA after ORIF failure; instead, outcomes were analyzed in terms of implant stability and functional recovery.

Postoperative management followed a standardized institutional protocol that remained unchanged throughout the study period. All patients received thromboprophylaxis with low-molecular-weight heparin for six weeks plus compression stockings, unless contraindicated. Indomethacin (75 mg/day for two weeks) was prescribed for prophylaxis against heterotopic ossification, reflecting institutional practice and supported by prior acetabular fracture reports.

Partial weight-bearing was initiated at six weeks and progressed to full weight-bearing by 12 weeks depending on construct stability and radiographic healing. Adherence was monitored through inpatient medication charts and outpatient interviews.

Patients were followed at six weeks, three months, six months, one year, and annually thereafter for a minimum of 24 months. At each visit, clinical and radiographic assessments were performed, and complications such as heterotopic ossification (HO), avascular necrosis (AVN), infection, implant failure, nerve injury, or post-traumatic arthritis were systematically documented.

Follow-up completeness was prospectively monitored. Time to secondary procedures (e.g., conversion to THA, HO excision, infection-related reoperation) was recorded in months from index surgery.

### Statistical analysis

Statistical analysis was performed using SPSS version XX (IBM Corp., Armonk, NY, USA). Continuous variables were summarized as means with standard deviations (SD) or medians with interquartile ranges (IQR), depending on normality tested by the Shapiro–Wilk test. Categorical variables were expressed as absolute frequencies and percentages.

Comparisons between categorical variables (e.g., fracture type vs. outcome category) were assessed using the Chi-square test or Fisher’s exact test where appropriate. For continuous outcomes, independent t-tests or Mann–Whitney U tests were employed depending on distribution. Correlations between surgical delay and outcomes were assessed using Pearson’s or Spearman’s correlation coefficients as appropriate.

To adjust for potential confounding factors, multivariable logistic regression models were constructed with functional outcomes (good/excellent vs. fair/poor) and radiological outcome (anatomical/imperfect vs. poor) as dependent variables. Candidate covariates included age, sex, fracture type, delay to surgery, and surgical approach. Model assumptions—including linearity in the logit, absence of multicollinearity, and overall goodness-of-fit by the Hosmer–Lemeshow test—were verified.

For secondary analysis, the cohort was stratified into two subgroups according to delay interval: early-late presentation (3–5 weeks after injury) and very-late presentation (> 5 weeks). This dichotomization was performed to explore whether the degree of surgical delay beyond the inclusion threshold (3 weeks) influenced outcomes. Between-group comparisons were conducted for radiological reduction quality, functional scores, complication rates, and secondary procedures, using Chi-square or Fisher’s exact tests for categorical variables and t-tests or Mann–Whitney U tests for continuous variables. This subgrouping allowed assessment of relative outcomes in moderately versus markedly delayed presentations, while maintaining consistency with the study’s original inclusion criteria.

Missing data were minimal (< 5% across all variables) and were handled by complete case analysis. A two-tailed p-value of < 0.05 was considered statistically significant. No formal correction for multiple comparisons was applied, as analyses were hypothesis-driven and limited in number. No a priori sample size or power analysis was performed, as the study represents the complete cohort of neglected acetabular fractures treated at our institution during the study period; this limitation has been explicitly acknowledged in the Discussion.

## Results

**Fig. 1:**
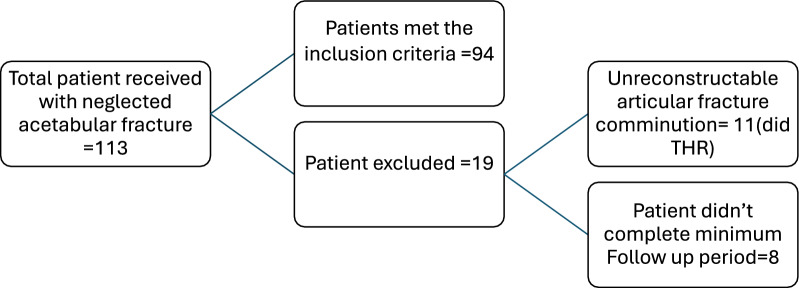
Flow diagram of patient selection. A total of 113 patients with neglected acetabular fractures were assessed. Nineteen patients were excluded (11 due to unreconstructable comminuted articular fractures treated with total hip replacement, and 8 due to incomplete minimum follow-up). Ninety-four patients met the inclusion criteria and were included in the final analysis

During the study period, 113 patients with displaced, neglected acetabular fractures were evaluated for surgical management. Eleven patients were deemed unsuitable for reconstructive fixation because of severe comminution, non-reconstructable articular surfaces, or advanced femoral head avascular necrosis; these patients underwent primary total hip arthroplasty (THA) and were excluded from the analytic cohort. Eight patients did not complete the minimum follow-up period of 2 years and were excluded. The remaining 94 patients underwent open reduction and internal fixation (ORIF) and form the basis of this report (Fig. 1 Flow diagram of patient selection). The mean follow-up duration among the ORIF cohort was 5.2 years (range: 2–6 years). Follow-up completeness at 2 years was 100% (94/94 patients), while at 5 years, 14 patients (14.9%) were available.

Among the 94 ORIF patients, there were 64 males (68.1%) and 30 females (31.9%), with a mean age of 38.7 years (range: 14–69). The right hip was affected in 52 cases (55.3%) and the left in 42 (44.7%). Road traffic accidents accounted for 68 cases (72.3%), fall from height for 21 (22.3%), and direct trauma for 5 (5.4%).

Fracture classification according to the Letournel–Judet system showed: both-column fractures in 24/94 (25.5%), posterior wall in 18/94 (19.1%), transverse with posterior wall in 15/94 (16.0%), posterior column with wall in 10/94 (10.6%), T-type in 8/94 (8.5%), anterior column in 7/94 (7.4%), posterior hemitransverse in 6/94 (6.4%), and complex associated pelvic/acetabular injuries in 6/94 (6.4%).

The mean delay to surgery was 36 days (range: 24–74). Mean operative time was 212 min (range: 160–320) and mean intraoperative blood loss was 930 mL (range: 500–1800).

By the final follow-up, all patients who retained their native hip after ORIF (84/94; 89.4%) demonstrated radiographic union with maintained acetabular dome continuity and no progressive displacement. Among the 10/94 (10.6%) who subsequently underwent THA, union of the native acetabulum was not assessable or applicable and these cases were excluded from the radiographic union analysis; instead, radiographs confirmed stable implant fixation and satisfactory cup positioning.

**Fig. 2:**
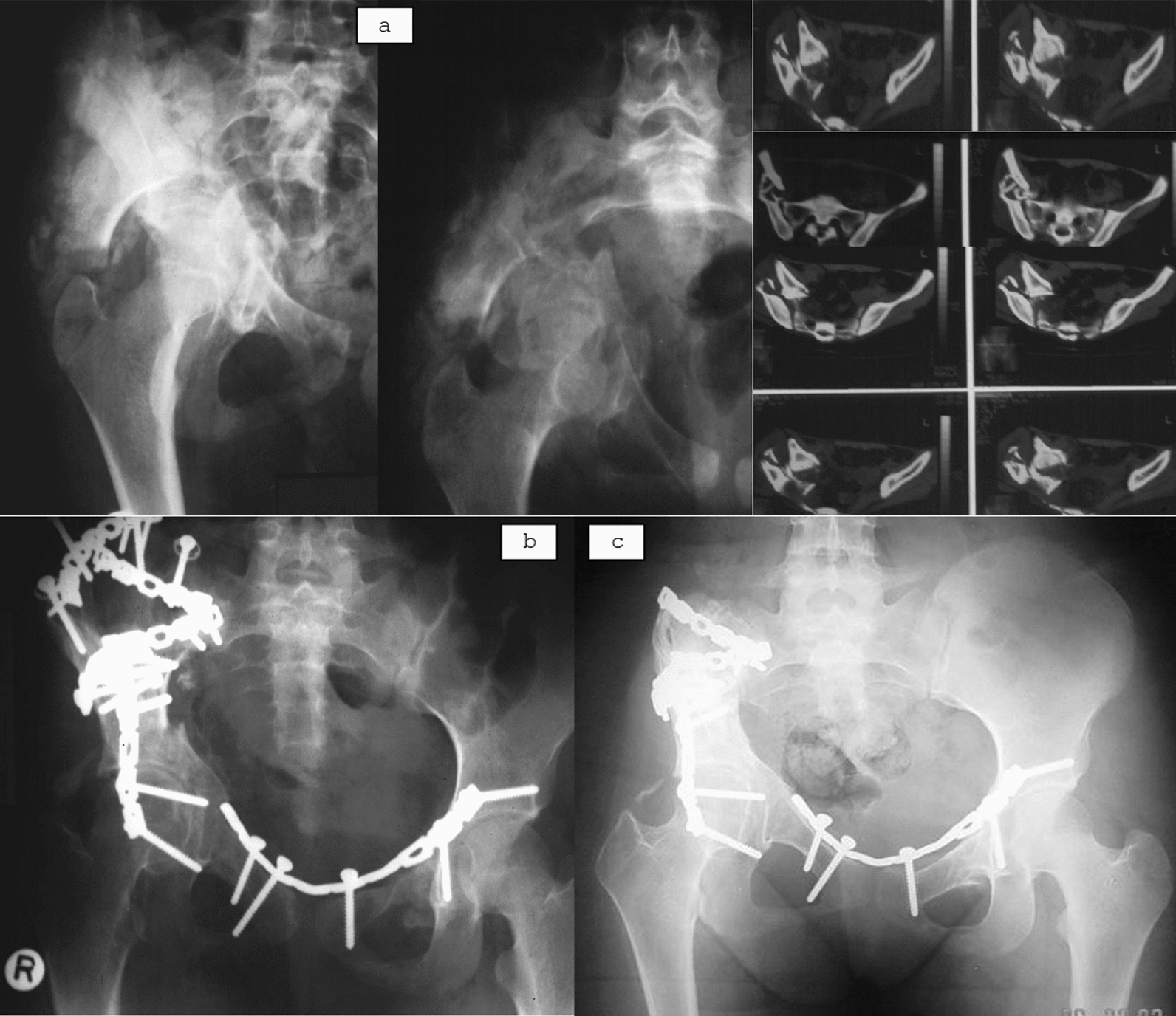
A 14-year-old girl with an 8-week-old right neglected associated both-column fracture. **a** Preoperative radiograph and Axial CT image showing severely displaced fracture with central femoral head subluxation and early callus formation. **b** 1 year follow up radiograph showing anatomical reduction and stable fixation with a congruent hip joint. **c** Final 6-year follow-up AP radiograph demonstrating excellent joint remodeling, maintained reduction, and implant removal around the iliac crest

**Fig. 3:**
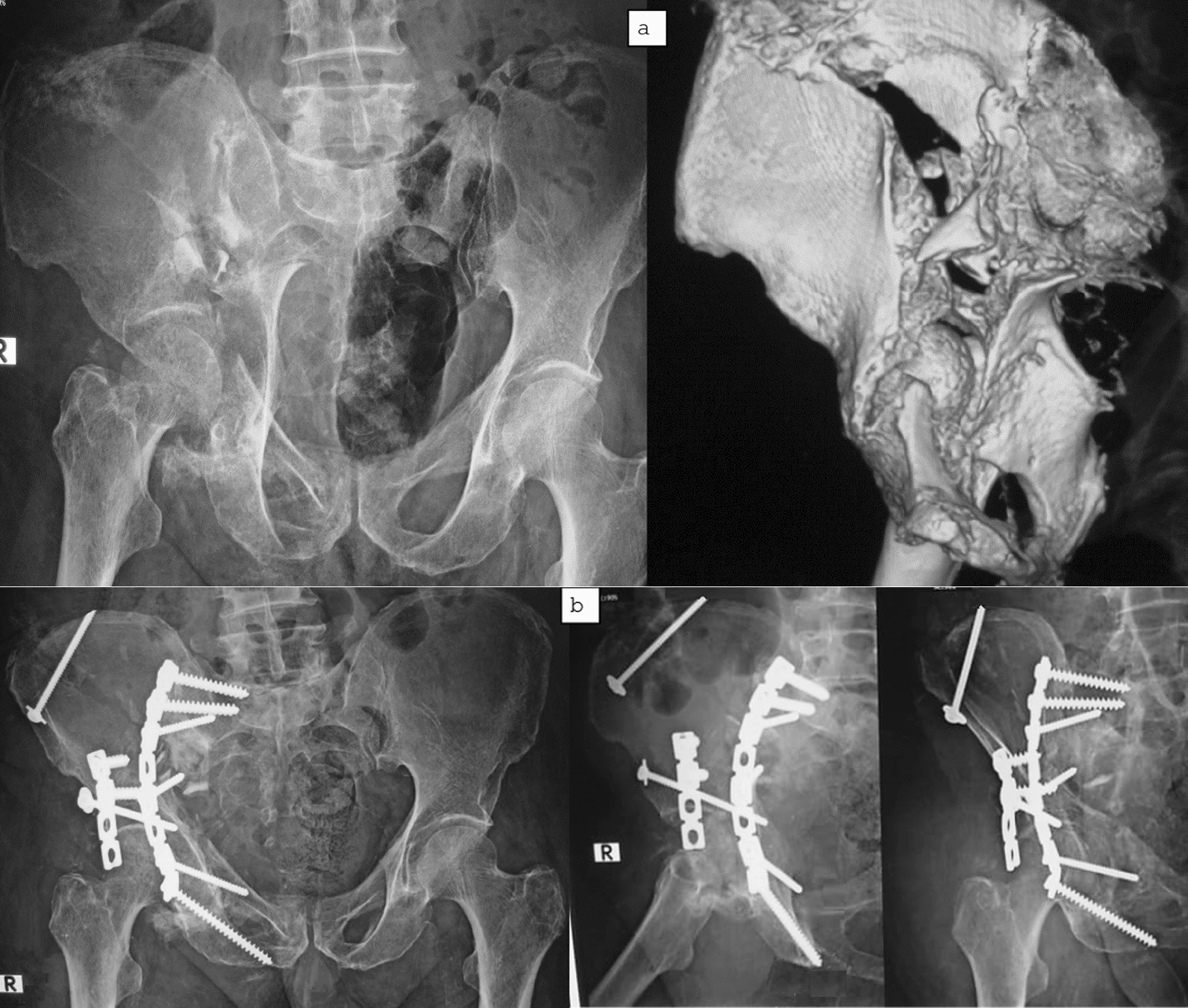
A 36-year-old male with a 5-week-old neglected both-column acetabular fracture. **a** Preoperative radiograph 3D CT showing displaced comminuted fracture with early callus. **b** Postoperative radiograph at the final follow-up visit showing acceptable reduction and fixation, with restoration of hip congruity

**Fig. 4:**
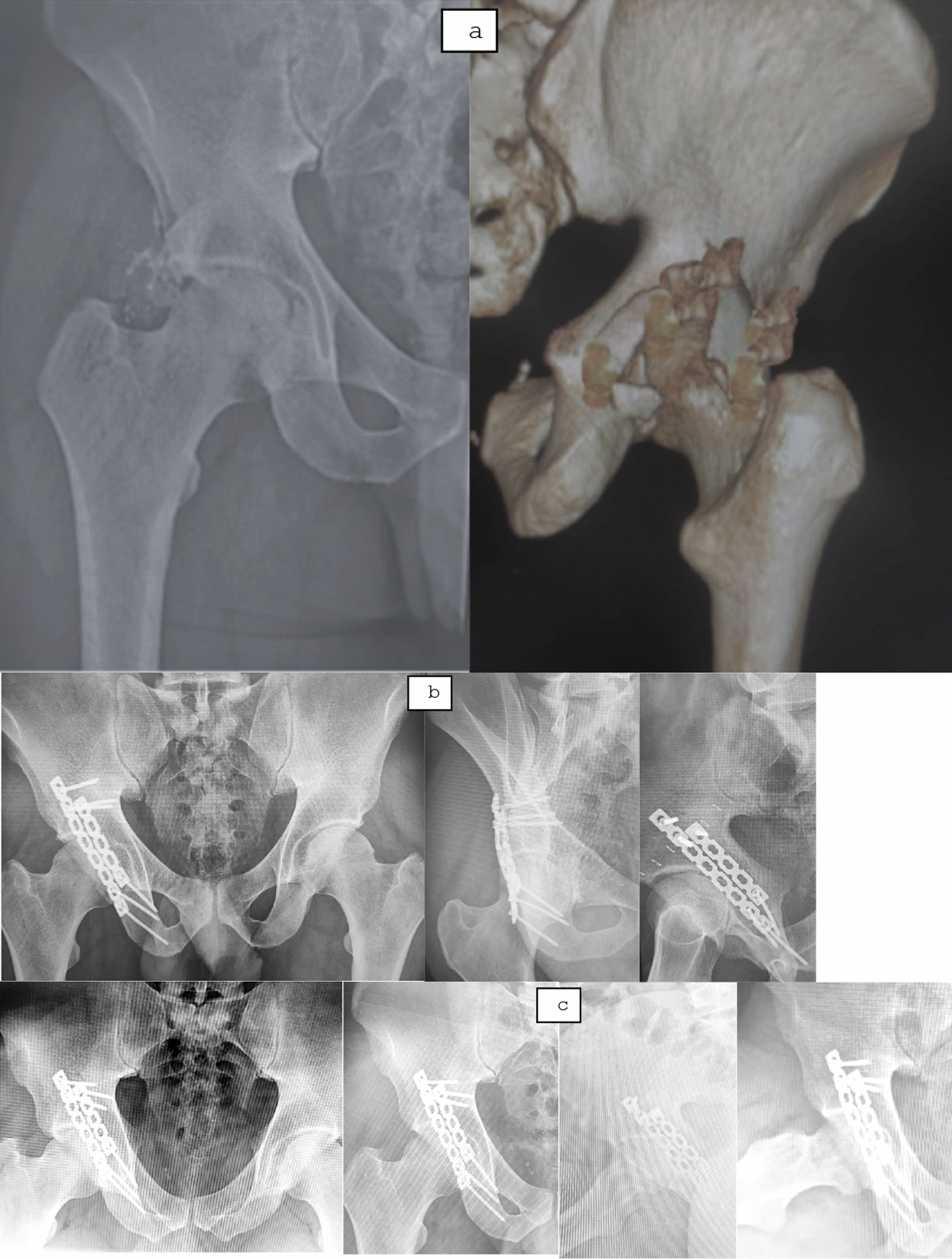
A 24-year-old male with a 6-week-old right comminuted posterior wall fracture. **a** Preoperative radiograph and 3D CT showed comminuted fracture with early callus formation. **b** Postoperative radiograph showing anatomical reduction with stable fixation. **c** Final 4 -year follow up radiograph showing maintained reduction with congruent hip

According to Matta’s criteria, 56/94 (59.6%) achieved anatomical reduction (≤ 1 mm), 25/94 (26.6%) imperfect (2–3 mm), and 13/94 (13.8%) poor (> 3 mm). Interobserver reliability was substantial for Matta’s grading (κ = 0.72) (Figs. 2, 3, 4 are case examples).

Functional outcomes, evaluated with the modified Merle d’Aubigné and Postel score, were excellent in 26 patients (27.7%), good in 41 (43.6%), fair in 17 (18.1%), and poor in 10 (10.6%). Patients who subsequently required conversion to THA were classified as poor outcomes in the primary analysis, reflecting failure of hip preservation. Interobserver agreement for functional scoring was excellent (ICC = 0.84), with disagreements resolved by consensus (Table 1).

**Table 1:** Demographics, injury characteristics, surgical details, and outcomes (n = 94)

Category	Details
Sex	Male: 64 (68.1%) Female: 30 (31.9%)
Mean age	38.7 years (range: 14–69)
Side of injury	Right: 52 (55.3%) Left: 42 (44.7%)
Mechanism of injury	Road traffic accident: 68 (72.3%), Fall from height: 21 (22.3%), Direct trauma: 5 (5.4%)
Mean time to surgery	36 days (range: 24–74 days)
Fracture type *(Letournel–Judet)*	Both-column: 24 (25.5%), Posterior wall: 18 (19.1%), Transverse + posterior wall: 15 (16.0%), Posterior column + wall: 10 (10.6%), T-type: 8 (8.5%), Anterior column: 7 (7.4%), Posterior hemitransverse: 6 (6.4%), combined pelvis/acetabulum: 6 (6.4%)
Mean operative time	212 min (range: 160–320)
Mean blood loss	930 mL (range: 500–1,800)
Intraoperative complications	2 patients (2.1%)—iatrogenic vascular injury
Radiographic outcome *(Matta criteria)*	Anatomical (≤ 1 mm): 56 (59.6%) Imperfect (2–3 mm): 25 (26.6%), Poor (> 3 mm): 13 (13.8%)
Functional outcome *(Modified Merle d’Aubigné and Postel score)*	Excellent: 26 (27.7%), Good: 41 (43.6%), Fair: 17 (18.1%), Poor: 10 (10.6%)
Mean follow-up duration	5.2 years (range: 2–6 years)
Postoperative complications	HO: 12 (12.8%), post-traumatic arthritis: 14 (14.9%), AVN: 9 (9.6%), Infection: 4 (4.3%), Nerve injury: 5 (5.3%)
Secondary procedures	THA conversion: 10 (10.6%) HO excision: 5 (5.3%)
Survival analysis	5-year native hip survival: 84% (95% CI 76–91), with conversion to THA as endpoint

To assess the impact of this definition, a sensitivity analysis was performed in which patients’ pre-THA functional scores were retained. Under this approach, the outcome distribution changed minimally (excellent/good: 71.3% vs. 72.3%; fair: 17.0% vs. 16.0%; poor: 11.7% vs. 11.7%), and the significance of associations between reduction quality, surgical delay, and functional outcomes remained unchanged. This indicates that the study findings are robust regardless of outcome classification strategy.

There was a strong positive correlation between reduction quality and functional scores (r = 0.48, *p* < 0.01). In contrast, fracture type (*p* = 0.60) and initial femoral head condition (*p* = 0.10) showed no significant associations. Delay to surgery, considered as a continuous variable, was negatively correlated with both radiological reduction (r = − 0.42, *p* < 0.01) and functional outcome (r = − 0.39, *p* < 0.01).

In multivariable regression, quality of reduction (β = 0.44, 95% CI 0.22–0.66, *p* < 0.001) and surgical delay (β = − 0.32, 95% CI − 0.54 to − 0.09, *p* = 0.006) remained independent predictors of functional outcome. Age, fracture type, and operating surgeon were not significant.

To further explore the influence of timing within this delayed cohort, patients were stratified into early-late presenters (3–5 weeks, n = 50) and very-late presenters (> 5 weeks, n = 44). Between-group differences in functional outcomes did not reach statistical significance (*p* = 0.31). Comparative results are summarized in (Table 2). However, when surgical delay was analyzed as a continuous variable, longer time to surgery was significantly associated with worse radiological reduction (r = − 0.42, *p* < 0.01) and poorer functional scores (r = − 0.39, *p* < 0.01).

**Table 2:** Comparison of outcomes between early-late (3–5 weeks) and very-late (> 5 weeks) presentation groups

Variable	Early-late (3–5 weeks, n = 50)	Very late (> 5 weeks, n = 44)	*p* value
Mean surgical delay (days)	29.6 ± 4.1	44.3 ± 6.2	< 0.001
Anatomical reduction (Matta ≤ 1 mm)	32 (64.0%)	24 (54.5%)	0.32
Functional outcome (Merle d’Aubigné–Postel)			0.31
Excellent/good	37 (74.0%)	30 (68.2%)	
Fair	8 (16.0%)	9 (20.5%)	
Poor	5 (10.0%)	5 (11.3%)	
Conversion to THA	4 (8.0%)	6 (13.6%)	0.37
Complications	6 (12.0%)	8 (18.2%)	0.41

While categorical subgroup analysis (3–5 weeks vs. > 5 weeks) showed no significant difference (*p* = 0.31) regarding functional outcome, continuous analysis revealed that progressively increasing delay correlated with poorer radiological and functional outcomes (r = − 0.42 and − 0.39, respectively; both *p* < 0.01). This explains the apparent discrepancy and highlights the limitations of dichotomization

This apparent discrepancy between non-significant subgroup comparisons and significant continuous correlations reflects the limitations of dichotomization. Splitting patients into two relatively small groups (3–5 weeks vs. > 5 weeks) reduced statistical power and masked incremental differences. In contrast, continuous modeling of surgical delay captured the full gradient of effect and consistently demonstrated a negative impact on both radiological reduction and functional recovery. Thus, progressively increasing delay worsens outcomes, even though arbitrary cut-points may not always yield statistically distinct groups.

Intraoperative complications occurred in 2/94 (2.1%), both involving iatrogenic external iliac vein injury during anterior exposure. One was controlled with primary repair and transfusion of 2 units of packed red blood cells; the second was managed with venorrhaphy and transfusion of 4 units, with no subsequent thrombotic events. Neither patient required reoperation nor developed long-term sequelae.

Other postoperative complications included surgical site infection in 4/94 (4.3%)—two superficial, managed with 10–14 days of oral antibiotics (cephalexin without hospital readmission), and two deep (Staphylococcus aureus, Enterobacter cloacae) requiring debridement and intravenous antibiotics for 6 weeks (vancomycin ± cefepime), and hospital stays of 10 and 14 days, respectively. Neither has chronic osteomyelitis. Heterotopic ossification was observed in 12/94 (12.8%): Brooker grade II in 7, grade III in 3, and grade IV in 2; five (grade III–IV) underwent excision with symptomatic relief. Avascular necrosis of the femoral head developed in 9/94 (9.6%), diagnosed by MRI at a mean of 14 months (range: 8–24); four progressed to collapse requiring THA. Post-traumatic arthritis occurred in 14/94 (14.9%), typically after 2–3 years, and six required THA. Neurological deficits occurred in 5/94 (5.3%): three incomplete sciatic neuropraxias and two femoral palsies, all traction related. Recovery was complete in four, with mild residual sensory deficit in one.

Secondary procedures included conversion to THA in 10/94 (10.6%) at a mean of 34 months after index surgery (range: 18–60 months) and HO excision in 5/94 (5.3%) at a mean of 22 months (range: 14–38). No perioperative deaths occurred. No patients were lost to follow-up before 24 months. Kaplan–Meier survival analysis of the native hip, with conversion to THA as the endpoint, demonstrated 84% survival at 5 years (95% CI 76–91).

## Discussion

Neglected acetabular fractures, typically defined as fractures not treated surgically within three weeks of injury, pose substantial challenges for orthopedic surgeons worldwide. These delays often result from systemic healthcare limitations, including deficient referral pathways, specialist shortages, limited hospital capacity, and delayed clinical decision-making. Patient-specific factors such as medical unfitness, soft tissue complications (e.g., Morel-Lavallée lesions), osteoporosis, and financial constraints further contribute to delayed intervention [1, 2]. Additionally, inadequate initial imaging or the absence of advanced CT-based fracture classification complicates timely and accurate diagnosis, thus hindering early surgical management [3].

Once the acute treatment window is missed, fracture pathology evolves unfavorably. Persistent displacement leads to femoral head abrasion by the sharp fractured acetabular edges, causing cartilage damage and significantly raising the risk of avascular necrosis (AVN) [4]. Biological healing processes including callus formation, fibrosis, and malunion further complicate mobilization and anatomical reduction of fracture fragments. Posterior wall fractures are prone to fragment necrosis and resorption, making surgical reconstruction more challenging [5]. These pathological changes necessitate extensive soft tissue dissection during surgery, increasing the risk of nerve injury, surgical site infection, and heterotopic ossification (HO). Prolonged preoperative immobilization also predisposes patients to secondary complications such as pressure ulcers and thromboembolic events [6].

The results of our study underscore the potential for favorable long-term outcomes in patients with neglected acetabular fractures treated with delayed open reduction and internal fixation. Despite an average surgical delay of 36 days, nearly 60% of patients achieved anatomical reduction, and over 70% attained excellent or good functional outcomes. These findings are particularly significant given the complexity and chronicity of the injuries, which often involve malunited fragments, soft tissue contractures, and distorted surgical planes.

In our cohort, surgical delay demonstrated a negative correlation with both reduction quality and functional scores when assessed as a continuous variable, underscoring the technical challenges posed by progressive fracture healing and fibrosis. However, when patients were dichotomized into early-late presenters (3–5 weeks) versus very-late presenters (> 5 weeks), no statistically significant difference was observed. This suggests that while progressive delay continues to erode the likelihood of optimal outcomes, the effect is more gradual rather than determined by a strict temporal cutoff. Therefore, our results support the concept that earlier intervention within the neglected phase can facilitate better outcomes, but we acknowledge that the evidence is associative rather than definitively causal, and categorical timing alone may not fully capture this effect. In addition, the apparent discrepancy between the non-significant subgroup comparison and the significant continuous analysis reflects a well-recognized limitation of dichotomization in clinical research. Arbitrary cutoffs can obscure gradual dose–response relationships by reducing statistical power and oversimplifying complex biological processes. Our findings are consistent with prior reports [7, 9, 14] that emphasize the incremental impact of progressive surgical delay rather than a strict temporal boundary. Recognizing this nuance not only clarifies our results but also highlights the importance of continuous modeling when assessing prognostic factors in neglected acetabular fractures.

Complication rates, including heterotopic ossification (12.8%), post-traumatic arthritis (14.9%), and avascular necrosis (9.6%), were within acceptable limits and consistent with previous literature on complex acetabular injuries. The need for secondary procedures, particularly conversion to total hip arthroplasty (THA), reflects the natural disease progression in certain patients but does not detract from the overall viability of ORIF as a limb-salvaging and function-preserving strategy. Collectively, these findings reinforce the notion that, with careful patient selection, preoperative planning, and specialized surgical execution, delayed ORIF remains a valuable and effective treatment modality for neglected acetabular fractures.

Despite these formidable challenges, ORIF remains a viable and often successful treatment option in carefully selected cases of neglected acetabular fractures (Table 3). Early pioneering work by Letournel et al. demonstrated that anatomical reduction could be achieved in roughly 50% of neglected fractures, with about half of these patients reporting good functional outcomes, although some eventually developed radiographic osteoarthritis [7]. Johnson et al., evaluating a larger cohort of 188 patients, reported anatomical reductions in 57%, with 65.5% achieving good-to-excellent clinical results. The study highlighted that anterior and posterior column fractures yielded better outcomes compared to posterior wall fractures, which had poorer prognoses (~ 51%) [8].

**Table 3:** Outcomes of delayed surgery for complex acetabular fractures among literature

Study	No. of cases	Mean delay to surgery	Radiological outcome (anatomic reduction %)	Functional outcome	Follow-up duration	Complications
Letournel et al. [7]	88	> 21 days	~ 50%	~ 50% good; 20% with OA	Up to 10 years	AVN, sciatic palsy, HO, fragment resorption
Johnson et al. [8]	188	> 3 weeks	57%	65.5% good-to-excellent	Mean 6.5 years (9 months–30 years)	AVN (26), HO (55), sciatic palsy (20), infection (8)
Mayo et al. [9]	64	> 21 days	56%	42% good-to-excellent; 38% poor	4.2 years	HO (33), AVN, nerve injuries, OA
Cai et al. [10]	61	22–399 days	74%	38 excellent, 13 good, 6 fair	Mean 36 months	HO (28), AVN (3), sciatic palsy (4)
Dilogo et al. [11]	6	~ 540 days (avg. 18 months)	Not reported	1 excellent, 4 fair, 1 poor (HHS)	18 months	OA (3), infection (1)
Zha et al. [12]	7	Not specified	Not reported	Excellent in pediatric; poor in adults	76 months	Post-traumatic OA in adults
Kumar et al. [13]	24	> 21 days	Not reported	57% good-to-excellent	Variable	AVN (4), HO (4), infection (3)
Singh et al. [14]	40	Median 42 days	Better in elementary patterns	60% good-to-excellent	Mean 2 years	HO, AVN (noted in delayed group)
Gupta et al. [15]	36	Not specified	Variable; better with good bone stock	Favorable in younger patients	1–5 years	Scar tissue, surgical difficulty
Acharya et al. (EIFA Review) [1]	— (Review)	> 3 weeks	Anatomical reduction possible with EIFA	Good outcomes in selected younger patients	Literature review (1999–2019)	HO risk present but controlled
Jenkinson et al. (Review) [2]	— (Review)	> 14–21 days	Reduction quality significantly worsens with delay	Poorer outcomes & QoL beyond 2–3 weeks	Literature review	Inferior function with delay

EIFA, extended ilio-femoral approach; HO, heterotopic ossification; AVN, avascular necrosis; OA, osteoarthritis, Functional outcomes are most reported using Harris Hip Score (HHS) or Merle d’Aubigné and Postel score. Many studies confirm deteriorating outcomes with delays beyond 21 days, especially in complex or reconstructable fracture types

Mayo et al. reported somewhat less favorable outcomes, with anatomical reduction achieved in 56% of 64 patients but only 42% attaining good-to-excellent results. Their cohort demonstrated a significant incidence of HO, AVN, nerve or vascular injury, and late degenerative changes, reinforcing the impact of surgical delay on prognosis [9]. Similarly, Cai et al. described 61 cases treated with delayed ORIF (mean delay 22–399 days) and achieved anatomical reductions in 74% of patients, many of whom had good to excellent functional outcomes. However, complications including HO (28 cases), AVN (3 cases), and sciatic nerve palsies (4 cases) were frequently encountered [10].

The findings align with those from a recent review focusing on complex acetabular fractures managed through delayed ORIF using the extended ilio-femoral approach, which reported anatomical reduction rates between 50 and 75% and good-to-excellent functional outcomes in 40–65% of cases. However, the review emphasized that complication rates including HO (up to 30%), AVN (10–15%), and sciatic nerve injury remained high, especially in fractures involving the posterior wall and dome impaction [1]. This corroborates prior data and reinforces the technical burden posed by the extended approach, particularly in cases with callus formation and scar tissue.

Dilogo et al. presented six cases with an average delay of 18 months before surgery, reporting a wide range of functional outcomes based on Harris Hip Scores (HHS), from excellent to poor [11]. Zha et al. found that pediatric patients with posterior wall fractures generally had excellent outcomes following delayed ORIF, whereas adults exhibited poorer results and more pronounced degenerative progression [12]. Kumar et al. evaluated 24 neglected fractures and achieved good-to-excellent outcomes in 57% of cases, though AVN, HO, and infections remained significant challenges [13].

More recently, Singh et al. assessed 40 neglected acetabular fractures treated with ORIF and reported a 60% rate of good-to-excellent outcomes. They identified elementary fracture patterns as having better prognosis compared to complex or associated fracture types, while posterior wall involvement and surgical delays beyond six weeks were significant predictors of poor outcomes [14]. Gupta et al. corroborated these findings, stressing that younger age, good bone stock, and careful patient selection contributed to favorable results despite technical difficulties [15].

When interpreting our findings in the context of prior studies, it is important to critically appraise not just outcomes but also the heterogeneity of patient populations, fracture characteristics, and surgical methods across the literature. Much of the variability in reported outcomes reflects methodological and patient-related differences rather than surgical timing alone.It is important to note that the heterogeneity of reported outcomes across published series is strongly influenced by differences in patient selection, definitions of ‘neglect,’ fracture patterns included, and surgical approaches employed. For example, studies with shorter delays (e.g., < 6 weeks) or predominantly elementary fracture patterns typically report higher rates of anatomical reduction and good functional recovery [7, 10, 14], whereas series including highly complex or malunited fractures, longer delays (> 3 months), or extended surgical approaches show less favorable outcomes and higher complication rates [9, 13]. Moreover, variability in functional scoring systems, follow-up duration, and reporting bias further complicate direct comparison. Taken together, these factors suggest that differences in study populations and methodologies, rather than surgical timing alone, may account for the wide range of outcomes described in the literature.

Surgical approaches chosen for neglected acetabular fractures depend largely on fracture pattern, delay duration, and the presence of fibrosis and callus. The extended ilio-femoral approach is frequently employed for complex fractures involving both columns or posterior wall, offering wide exposure essential for thorough debridement and mobilization of malunited fragments [1]. The anterior approach, including the modified Stoppa technique, can be effective in selecting anterior column fractures or those with limited displacement, reducing soft tissue morbidity [15, 16]. Surgeons often combine approaches to access multiple fractured planes, especially when dealing with extensive scarring and distorted anatomy.

Successful reduction hinges on several intraoperative strategies. Meticulous soft tissue dissection to release contracted tissues and remove callus is crucial to restore mobility of fracture fragments. Use of Schanz pins or temporary external fixators as “joysticks” aids manipulation of fragments, while precontoured plates and clamps facilitate provisional stabilization [16, 17, 18]. Judicious use of cortical windowing and bone grafting helps address bone defects and supports fixation, particularly in cases with osteopenia or fragment resorption [23]. Intraoperative fluoroscopy and 3D imaging guidance have enhanced accuracy in fragment alignment and implant positioning, minimizing residual displacement.

Fixation techniques vary depending on fracture morphology and bone quality. Locking plates, pelvic reconstruction plates, and spring plates are commonly utilized to achieve stable fixation allowing early mobilization. In complex fractures with dome impaction or comminution, supplemental structural bone grafting and lag screws improve stability and articular congruity [23]. Additionally, when bone stock is poor or articular damage severe, staged or primary total hip arthroplasty combined with ORIF (fix-and-replace technique) is increasingly adopted, particularly in older patients [19–22]. Postoperative protocols emphasize early mobilization balanced against protection of fixation to reduce complications such as HO and infection [1].

Advanced 3D CT imaging has revolutionized preoperative planning, particularly in neglected fractures, allowing detailed evaluation of fracture union, fragment viability, and surgical feasibility. Structural iliac crest bone grafting has been successfully used to reconstruct posterior wall defects, improving joint congruity and containment of the femoral head [23, 24].

This study represents one of the largest prospective series addressing displaced, neglected acetabular fractures with midterm follow-up that was conducted at a high-volume pelvic trauma center over a 10-year period where it benefits from standardized imaging, surgical protocols, and functional assessment using validated scoring systems. However, this study has several important limitations that warrant careful consideration. First, it is a single-center series, which may restrict the generalizability of findings. Although the cohort size is relatively large for neglected acetabular fractures, subgroup analyses were underpowered, limiting conclusions about specific fracture types or approaches. Second, no predefined statistical analysis plan was registered prior to the study, which introduces a potential risk of selective reporting and post hoc interpretation. Third, sample denominators were not always consistent across analyses due to missing data, which may bias certain comparisons. Finally, details of surgeon experience and the evolution of surgical techniques over the 10-year period were not fully captured; both may have influenced outcomes. These factors introduce risks of bias and confounding that should temper the interpretation of our findings, and they underscore the need for larger, multicenter, prospectively designed studies with standardized protocols.

## Conclusion

Our findings confirm that delayed open reduction and internal fixation (ORIF), even beyond the traditional acute window, remains a safe and effective treatment strategy in appropriately selected patients. With a multidisciplinary approach, the use of individualized surgical techniques including combined anterior and posterior approaches and adherence to standardized postoperative protocols, anatomical reduction was achieved in nearly 60% of cases, and more than 70% of patients attained good-to-excellent functional outcomes.

Importantly, earlier surgical intervention within the neglected phase correlated with significantly better radiological and functional results, highlighting the continued importance of timely referral and surgical planning even after the initial injury window has passed. Although complications such as heterotopic ossification, avascular necrosis, and post-traumatic arthritis were observed, their rates were consistent with or lower than those reported in similar literature.

In conclusion, delayed ORIF can provide durable, joint-preserving results in cases of neglected acetabular fractures. With careful patient selection, thorough preoperative planning, and experienced surgical execution, it remains a valuable alternative to salvage procedures or early arthroplasty—particularly in younger patients or those with reconstructable fracture patterns.

## Data Availability

The datasets used and/or analyzed during the current study available from the corresponding author on reasonable request.
